# Adaptive Radiotherapy for Carcinoma Endometrium With Lymphocele: A Case Report

**DOI:** 10.7759/cureus.65775

**Published:** 2024-07-30

**Authors:** Induni N Weerarathna, Ashish Uke, Manishimwe Jules, Shweta B Dahake, Anurag Luharia

**Affiliations:** 1 Biomedical Sciences, Datta Meghe Institute of Higher Education and Research, Wardha, IND; 2 Radiation Oncology, Datta Meghe Institute of Higher Education and Research, Wardha, IND; 3 Radiotherapy, Datta Meghe Institute of Higher Education and Research, Wardha, IND; 4 Medical Physics, Datta Meghe Institute of Higher Education and Research, Wardha, IND

**Keywords:** vaginal brachytherapy, adjuvant chemotherapy, high-grade endometrioid carcinoma, adaptive radiotherapy, endometrial carcinoma

## Abstract

The case report details the adaptive radiotherapy management of a 75-year-old female diagnosed with high-grade endometrial carcinoma. The patient, who was known to be hypertensive with no other comorbidities and no family history of cancer, presented with a complaint of bleeding per vagina for six months. Following extensive investigations, she underwent a laparoscopic radical hysterectomy. Postoperative histopathology confirmed endometrial adenocarcinoma International Federation of Gynecology and Obstetrics (FIGO) stage IA, grade III. The adjuvant treatment plan included adjuvant chemoradiotherapy to the postoperative tumor bed and draining lymph nodes. On planning computed tomography (CT), the patient’s lymphocele responded remarkably to radiation therapy, an unusual outcome that underscores the potential efficacy of adaptive radiotherapy in complex cases.

## Introduction

Gynecologic cancer, or GYN cancer for short, is cancer that starts in a woman's reproductive system. The most common GYN malignancies include tumors of the vulvar, vaginal, ovarian, uterine, and cervical regions. Gynecologic cancers afflict over 94,000 women in the US each year. The most common kind of GYN cancer is uterine cancer, which is followed by ovarian and cervical cancer [1]. Cervical cancer is the fourth most common cancer in the world for which women are diagnosed [2]. Treatment options for GYN cancers include radiation, chemotherapy, and surgery [3]. After surgery, adjuvant or final radiation is often given to patients with high-risk features or locally advanced diseases. In contrast, brachytherapy is a further form of radiation therapy that may be administered to others [4].

Adaptive radiation was first described in the 1990s by Yan et al. This publication's authors described a radiation therapy that could be modified as it was being administered in response to changes in anatomy [5]. For the treatment of locally advanced cervical cancer, cisplatin and concurrent external beam radiation therapy (EBRT) are frequently utilized in addition to brachytherapy. The term "adaptive radiation therapy" (ART) refers to methods that let a radiation therapy treatment plan change based on pictures obtained while the patient is receiving treatment. The switch to image-guided adaptive brachytherapy (IGABT) for cervical cancer demonstrates the benefits of this strategy [6]. Adaptive radiation can be very beneficial for GYN tumors for two main reasons. First, the size of GYN malignancies, including cervical cancer, can vary significantly after treatment. By taking these variations in tumor size into account, adaptive radiation treatment may be able to target less healthy tissue while still treating cancer [3].

In wealthy nations, endometrial carcinoma is the most prevalent type of gynecologic cancer. Depending on the stage and grade of the disease, adjuvant radiation therapy, chemotherapy, and surgery are usually the next steps in the treatment procedure [7]. To potentially improve outcomes and minimize side effects, ART can be adjusted to account for changes in the tumor's growth and the patient's anatomy throughout radiation therapy. This case study discusses the use of ART in a lady with grade III endometrial cancer, Federation of Gynecology and Obstetrics (FIGO) stage IA, compounded by the development of a postoperative lymphocele. Interestingly, radiation therapy is frequently ineffective for lymphoceles; nevertheless, in this instance, the lymphocele responded well, underscoring the possible advantages of antiretroviral therapy [8].

## Case presentation

A 75-year-old postmenopausal female with a known history of hypertension managed on medication and no significant family history of cancer presented with vaginal bleeding that began in December 2023. She consulted a doctor, and initial blood investigations were conducted, and the results came within normal limits. Subsequent screening for human immunodeficiency virus (HIV) was done and returned non-reactive results.

**Figure 1:**
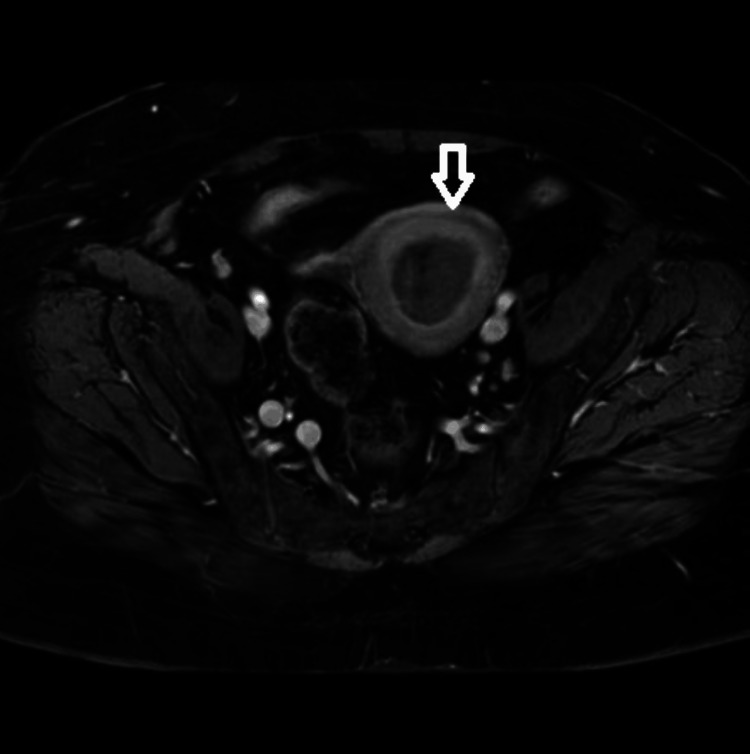
Axial T1-weighted contrast-enhanced MRI of the pelvis showing a lesion in the fundal wall of the uterus on the left side MRI: magnetic resonance imaging

**Figure 2:**
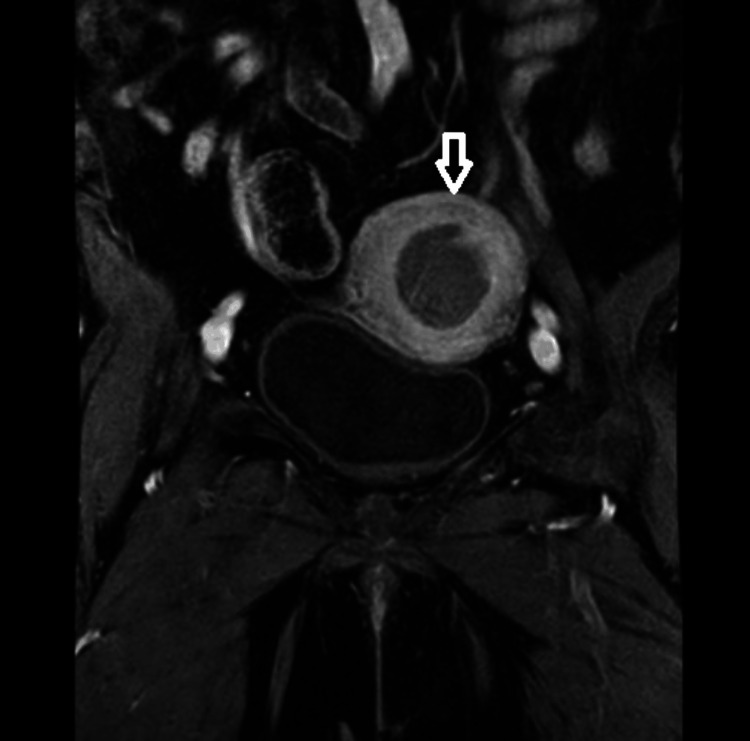
Coronal T1-weighted contrast-enhanced MRI image showing a lesion in the fundic region on the left side MRI: magnetic resonance imaging

For further evaluation, the patient was referred to a gynecologist. A magnetic resonance imaging (MRI) of the pelvis performed at the hospital revealed a bulky uterus measuring 6.0 × 6.9 × 9.2 cm with thickened endometrium showing heterogeneous enhancement, as shown in Figures 1-2. The tumor measured approximately 3.5 x 4.4 x 4.4 cm and involved the inner 50% of the myometrium in the fundic region. Additionally, a few sub-centimetric internal and external iliac lymph nodes on the left side appeared suspicious.

After proper preoperative investigations and a pre-anesthetic checkup, the patient underwent a laparoscopic radical hysterectomy with bilateral salpingo-oophorectomy and bilateral pelvic lymph node dissection performed by a surgical oncologist. The postoperative histopathology report confirmed endometrial adenocarcinoma, FIGO grade III. The specimen measured 10.0 x 9.0 x 6.5 cm, with a tumor size of 3.5 x 3.0 cm. The tumor infiltrated less than half of the myometrium thickness (18 mm), and the uterine serosa appears negative for tumor involvement. There was no involvement of the lower uterine segment or lymphovascular space invasion, and all dissected lymph nodes (pelvic and iliac: 0/10; right para-aortic: 0/2; total: 0/12) were negative for malignancy. Immunohistochemistry results showed positivity for p53 and vimentin.

The patient's postoperative clinical examination revealed good general condition, a Karnofsky Performance Status (KPS) of 80, and no signs of pallor or icterus. On pelvic examination, the vaginal length was measured at 6 cm with a diameter of 2-5 cm. The final diagnosis was FIGO stage IA, grade III endometrial carcinoma. Given the high-grade nature of the tumor and the risk of residual microscopic disease, the treatment plan included adjuvant chemoradiotherapy to the whole pelvis (post-operative tumor bed + draining lymph nodes) at a dose of 50 Grey (Gy) in 25 fractions using the intensity-modulated radiation therapy (IMRT) technique with concurrent Cisplatin.

The patient and her relative were counseled regarding the need for adjuvant radiation therapy, and they agreed to the same. The patient was called for a planning scan where she was simulated in a first supine position with arms overhead, supported with a headrest and knee rest, and immobilized with a pelvic four-clamp thermoplastic mask. The patient was instructed to drink 500 ml of water 45 minutes before the procedure. A contrast-enhanced planning scan was taken from the L2 vertebrae to the midthigh, with 2.5 mm cuts. The scan was imported to the SOMAVision contouring system, and contouring of the area of interest with proper registration with preoperative MRI pelvis was done according to the international consensus guidelines, including the preoperative gross tumor volume plus pelvic nodes as the CTVp and CTVn, and a planning target volume (PTV) margin of 5 mm was given to define a PTVp and PTVn. The organs at risk (OARs) were also delineated, including the bladder, rectum, sigmoid colon, femoral heads, and bowel bag.

**Figure 3:**
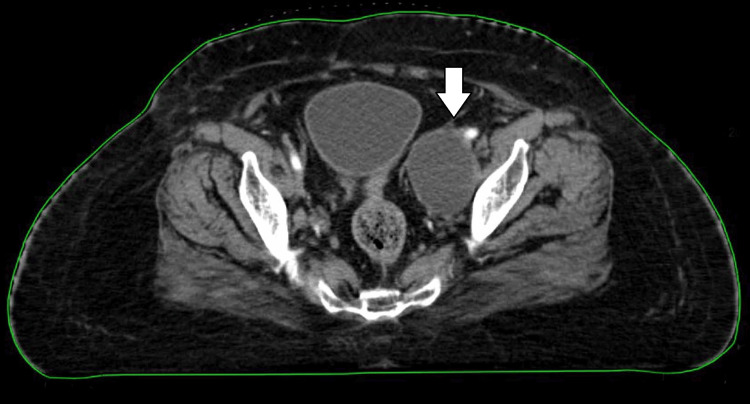
Planning CT showing large lymphocele at the pelvic region on the left side near the left external iliac vessel CT: computed tomography

During contouring on the planning CT, a large lymphocele was observed on the left side in the pelvic region near the left external iliac region with a volume of 69.2 cc, as shown in Figure 3. A surgical oncologist's opinion regarding surgery was taken, and due to advanced age and associated comorbidities, surgery was deferred for the patient. For the sake of benefit, the radiation oncologist planned to include lymphocele in the target volume and assessment after 10 fractions of radiotherapy were planned for the need for adaptive radiotherapy planning. The patient was started on radiation therapy treatment with five fractions per week, along with concurrent chemotherapy with weekly Cisplatin. The patient was assessed weekly for acute skin, gastrointestinal, and genitourinary toxicities.

For the treatment, an inverse IMRT plan was made on Varian Eclipse version 16.1 using seven fields for a dose of 50 Gy in 25 fractions. PTV's upper and lower constraints were met, and the plan was optimized for the best OAR doses possible. The D_max_ for the plan was 104.4%, the 3D mean dose was 99.5%, and the 3D min dose was 84.5%. About 95% of the dose was received by 96.5% PTV volume. The OAR doses were as follows: Bladder D_max_ = 51.84 Gy and V20 Gy was 50.14 Gy; femoral head left and right D_max_ = 48.8 Gy and 48.7 Gy; sigmoid D_max_ = 52.19 Gy; rectum D_max_ = 51.24 Gy; bowel D_mean_ = 16.742 Gy and V20 Gy was 50.0 Gy.

**Figure 4:**
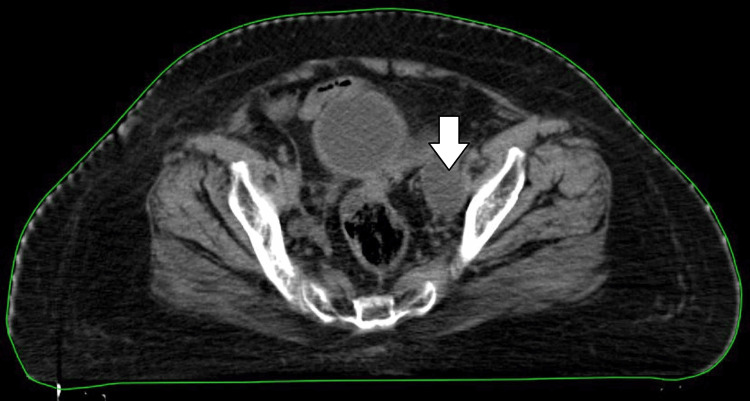
Post 10 fractions adaptive planning CT showing significant lymphocele volume reduction CT: computed tomography

On serial cone beam CT for daily image-guided radiation therapy (IGRT), gradual regression of the lymphocele was observed, and a planning scan of 10 fractions of EBRT was performed for adaptive planning. Significant regression of lymphocele was observed (post 10 fractions RT volume was 23.1 cc, as shown in Figure 4.

The adaptive contour was drawn along with the respective OARs, and adaptive planning was performed for the remaining 15 fractions of external beam radiotherapy. Some doses for both plans, along with OARs doses, are mentioned in Table 1.

**Table 1:** Plan sum doses for original and adaptive plans OARs: organs at risk; Gy: gray

Target and OARs	Original plan (10 fractions)	Adaptive plan (remaining 15 fractions)	Plan sum doses
Rectum (D_max_)	20.49 Gy	30.49 Gy	50.98 Gy
Bladder (D_max_)	20.7 Gy	30.67 Gy	51.37 Gy
Sigmoid (D_max_)	20.8 Gy	31.02 Gy	51.82 Gy
Femoral head left (D_max_)	19.5 Gy	28.98 Gy	48.48 Gy
Femoral head right (D_max_)	19.4 Gy	28.78 Gy	48.18 Gy
Bowel (D_mean_)	6.7 Gy	13.11 Gy	19.81 Gy

**Figure 5:**
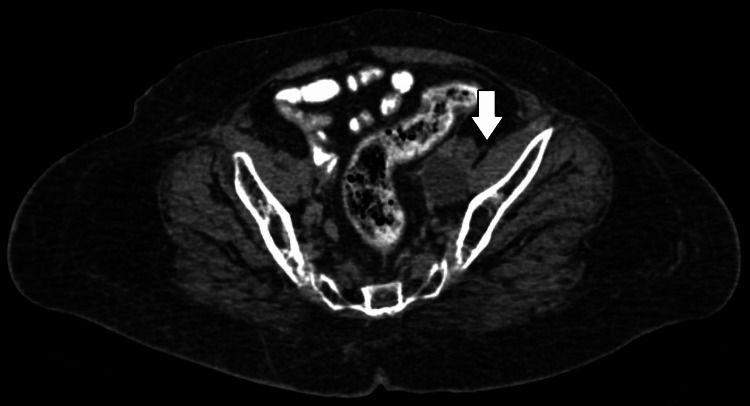
Response CT showing further reduction of lymphocele CT: computed tomography

After the completion of radiotherapy, the patient was discharged and called for a toxicity assessment after 15 days of radiotherapy. Response scans post-radiotherapy showed progressive and significant lymphocele reduction, as shown in Figure 5 (post-radiotherapy lymphocele volume 16.7 cc), and radiotherapy as an alternative and effective treatment option.

## Discussion

The benefits of incorporating a patient's position variation into the treatment optimization process during radiotherapy were initially highlighted by Yan et al. in their description of the role of ART. This procedure can be carried out effectively in radiation clinics nowadays by incorporating cutting-edge technologies. The ART procedure can improve radiation therapy by altering the treatment dose and margin according to each patient's unique variation [5].

Adaptive radiation can be very beneficial for GYN malignancies for two main reasons. First, the size of GYN tumors can alter significantly throughout treatment, much like it can with cervical cancer [9]. These variations in tumor size can be accommodated by adaptive radiation, which may also allow for the treatment of less healthy tissue while preserving tumor coverage. The bladder, rectum, and bowel are located in the pelvis and can migrate dramatically between treatment portions. Once more, adaptive radiation can cure less normal tissue and explain this interfraction motion [3].

Jereczek-fossa et al. conducted a tiny study titled "Radiotherapy in prostate cancer patients with pelvic lymphocele." Following surgery, pelvic lymphocele was found on the simulated CT scan in 30 of 308 patients (10%) who received radiation therapy following prostatectomy. Clinical and dosimetric data were assessed between January 2011 and July 2013. The median lymphocele volume was 47 cm^3^ (range: 6 to 467.3 cm^3^). In eight cases (27%), lymphoceles were left out of the PTV. There was a maximum dose of 57 Gy (5.7-73.3 Gy) for lymphocele. They concluded that radiotherapy following prostatectomy is doable with manageable acute and late effects when there is pelvic asymptomatic lymphocele. The lymphocele volume shrank during radiation therapy, necessitating the revision of the intermediate radiotherapy plan [10].

The capacity of ART to modify treatment regimens in response to anatomical changes and real-time imaging has demonstrated a great deal of promise for improving treatment results and precision. This example supports ART's broader use in complicated gynecologic oncology situations, in line with results by Wang et al. that ART can minimize toxicity and lower PTV margins in endometrial and cervical cancer. More studies are required to examine and optimize ART methods and their potential to improve patient outcomes fully [11].

Adjuvant chemoradiotherapy and vaginal brachytherapy, together with ART, were combined to give a comprehensive treatment plan for this instance of high-grade endometrial cancer. The multidisciplinary strategy, which combined cutting-edge technologies with established therapy processes, allowed for the best potential therapeutic outcome [12]. Like Thörnqvist et al.'s investigation of ART procedures for pelvic tumors and their finding of improved dosimetric outcomes with the treatment, this case supports the broader use of ART in complex gynecologic oncology circumstances [13].

## Conclusions

The management of challenging instances of high-grade endometrial cancer with ART is demonstrated by this case, particularly when postoperative complications like lymphocele complicate the treatment. Despite its typical resistance to radiation therapy, the lymphocele responded favorably to ART, demonstrating the effectiveness of ART in adjusting to anatomical changes and improving treatment outcomes. This case suggests that real-time modifications to radiation therapy can significantly improve accuracy and efficacy, which supports the broader use of ART in gynecologic oncology. More investigation into its mechanics and advantages is necessary to establish ART's place in cutting-edge cancer therapy protocols.
